# Cyst in the Orbital Cavity

**Published:** 1844-06

**Authors:** 


*Cyst in the orbital cavity.* By Dr. Dornbluth.—A girl, 22 years of age, was seized with a rheumatic affection of the eye, for which antiphlogistic remedies were employed. These relieved the symptoms, but in a few weeks the eyelids swelled, the eye became red and painful, and resisted all modes of cure. About four months afterwards she consulted Dr. Dornbluth. The pain in the eye was then so acute as to prevent sleep, and her body had wasted considerably. The eye was pushed out of its situation towards the temporal region, so as to be beyond the orbit. The eyelid, though stretched to its utmost extent, could not cover more than the half of the cornea; which, however, still retained its transparency. The iris was dilated and immovable, but the anterior chamber of the eye appeared natural. The most attentive examination could not detect the slightest alteration in the deep humors of the eye. Vision had been failing for about four months, but now the brightest sun's rays produced no effect on the eye; there was total blindness. The sclerotic conjunctiva was very red, swollen and resistant, and formed a considerable projection. Behind and on the nasal side of the eyeball, projected a fleshy mass, of about the size of an egg, and extending from the middle of the nasal to the superior maxillary bone. This fleshy mass was exquisitely tender, was covered with a smooth membrane, and discharged, copiously, mucous fluid and tears. The girl's health was seriously affected; she was feverish, had thirst, and loss of appetite.

As the nature of the tumor was not at first recognized, and topical applications gave no relief, a puncture was made in it, from which a considerable quantity of transparent serous-looking fluid escaped, and continued more or less for two days, by which time a notable diminution of its size was observed. The aperture was then enlarged, and it was recognized that the tumor consisted of a cyst about two lines in thickness, and two and one-half inches in depth, which filled the whole of the orbital cavity, and pushed the eyeball forwards. The cavity of the cyst was filled with lint, an abundant suppuration ensued, and the fifth day thereafter the membrane of the cyst was thrown off. The tumor diminished rapidly in size after this, and the wound healed over in eight days. The eyeball regained its normal position, and was again covered by the eyelid, which moved freely over it, but the eye never recovered its sensibility to light.—*Edin. Med. & Surg. Jour—Am. Jour. Med. Sci.*

